# Intra‐ and interspecific variation in spectral properties of dominant *Sphagnum* moss species in boreal peatlands

**DOI:** 10.1002/ece3.10197

**Published:** 2023-06-13

**Authors:** Sini‐Selina Salko, Jussi Juola, Iuliia Burdun, Harri Vasander, Miina Rautiainen

**Affiliations:** ^1^ School of Engineering Aalto University Espoo Finland; ^2^ Department of Forest Sciences University of Helsinki Helsinki Finland

**Keywords:** boreal, hyperspectral, moisture, reflectance, remote sensing, spectrum

## Abstract

Boreal peatlands store ~25 % of global soil organic carbon and host many endangered species; however, they face degradation due to climate change and anthropogenic drainage. In boreal peatlands, vegetation indicates ecohydrological conditions of the ecosystem. Applying remote sensing would enable spatially and temporally continuous monitoring of peatland vegetation. New multi‐ and hyperspectral satellite data offer promising approaches for understanding the spectral properties of peatland vegetation at high temporal and spectral resolutions. However, using spectral satellite data to their fullest potential requires detailed spectral analyses of dominant species in peatlands. A dominant feature of peatland vegetation is the genus *Sphagnum* mosses. We investigated how the reflectance spectra of common boreal *Sphagnum* mosses, collected from waterlogged natural conditions after snowmelt, change when the mosses are desiccated. We conducted a laboratory experiment where the reflectance spectra (350–2500 nm) and the mass of 90 moss samples (representing nine species) were measured repetitively. Furthermore, we examined (i) their inter‐ and intraspecific spectral differences and (ii) whether the species or their respective habitats could be identified based on their spectral signatures in varying states of drying. Our findings show that the most informative spectral regions to retrieve information about the *Sphagnum* species and their state of desiccation are in the shortwave infrared region. Furthermore, the visible and near‐infrared spectral regions contain less information on species and moisture content. Our results also indicate that hyperspectral data can, to a limited extent, be used to separate mosses belonging to meso‐ and ombrotrophic habitats. Overall, this study demonstrates the importance of including data especially from the shortwave infrared region (1100–2500 nm) in remote sensing applications of boreal peatlands. The spectral library of *Sphagnum* mosses collected in this study is available as open data and can be used to develop new methods for remote monitoring of boreal peatlands.

## Graphical abstract

We measured the spectral properties of boreal peatland *Sphagnum* species. The most informative spectral regions to retrieve information about the *Sphagnum* species and their state of desiccation are in the shortwave infrared region. Our results can be used to develop methods for remote monitoring of boreal peatland vegetation.

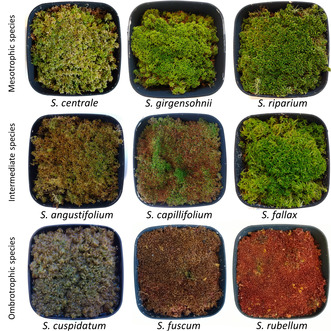

## 1 INTRODUCTION

Northern peatlands have a critical role in the global carbon cycle and climate system (FAO, 2020; Harenda et al., 2018). Constituting up to 25% of soil organic carbon (Loisel et al., 2021; Yu et al., 2010), they have functioned as a strong sink and stock of organic carbon throughout the Holocene (Nichols & Peteet, 2019). They are also hotspots for biodiversity (Fraixedas et al., 2017; Saarimaa et al., 2019) and can store large quantities of water, making them important water‐retaining and water‐regulating ecosystems (Blodau & Moore, 2003).

The ecohydrology of a peatland site, that is, the spatial, temporal, and qualitative variation of the incoming water on the site, determines the structure and species composition of the site's vegetation communities (Laine & Vasander, 1996). An established way to classify peatlands is according to their ecohydrological qualities into ombrotrophic and minerotrophic and further into eu‐, meso‐, and oligotrophic peatlands (Rydin & Jeglum, 2006). Sites referred to as minerotrophic receive their water from nutritious mineral soil and surface water as well as from precipitation and thus, have a higher nutrient availability than the ombrotrophic sites. Sites with a thick enough peat layer to isolate the surface vegetation from the nutritious mineral soil water, making precipitation their only source of water are called ombrotrophic. Sub‐categories of minerotrophic sites, eu‐, meso‐, and oligotrophic sites form a sequence of nutrient levels with decreasing nutrient availability.

One of the predominant features of the vegetation of boreal peatlands is the genus *Sphagnum* mosses, also known as peat mosses (Rydin et al., 2006). Because of their substantial biomass production rate as well as relatively slow decay rate (Limpens & Berendse, 2003), *Sphagnum* mosses are the leading component of peat formation in boreal peatland ecosystems (Robroek et al., 2007). They are also commonly used as indicators of a peatland site's ecohydrology because of their narrow habitat preferences (Johnson et al., 2015). Thus, remote monitoring of *Sphagnum* mosses and changes in their coverage is critical for understanding how the vegetation and growth conditions in peatlands are developing due to climate change and anthropogenic drainage.

Compared with vascular plants, the spectral characteristics of mosses, and especially the genus *Sphagnum* mosses, have been found to differ and have very distinctive spectral characteristics in visible (VIS, 400–700 nm), near‐infrared (NIR, 700–1300 nm) and shortwave infrared (SWIR, 1300–2500 nm) regions (Bubier et al., 1997; Vogelmann & Moss, 1993). Several studies have also documented the high sensitivity of *Sphagnum* spectra to moisture (Bryant & Baird, 2003; Harris et al., 2005; Lees et al., 2019; Van Gaalen et al., 2007). As a bryophyte, *Sphagnum* is sensitive to changes in moisture availability (Bragazza, 2008; Gerdol et al., 1996), and undergoing water stress results in the loss of the plant's pigmentation. However, different *Sphagnum* species can tolerate different amounts of water stress before beginning to appear whitish (Bragazza, 2008; Koks et al., 2022).

Hyperspectral satellite data that will be increasingly available from new and forthcoming satellite missions (e.g., EnMAP, PRISMA, CHIME) will open novel possibilities for monitoring peatland vegetation at the species level due to the higher spectral coverage. However, to use the novel hyperspectral airborne or satellite data sets to their full spectral extent, detailed measurements of the spectral properties of key plant species are needed. The potential for using spectral data to differentiate *Sphagnum* species from each other has been speculated in previous literature, as the spectral attributes of different *Sphagnum* species have been found to be remarkably different from each other in laboratory and field measurements (Harris & Bryant, 2009; Tucker et al., 2022; Vogelmann & Moss, 1993). Nevertheless, intraspecific variation in *Sphagnum* species' spectra remains poorly understood, both because the sample sizes in previous studies have been very small, often only one sample per species, or the number of studied species has been very limited. Furthermore, nearly all recent research on *Sphagnum's* spectral characteristics has been conducted in temperate peatlands (Bryant & Baird, 2003; Harris, 2008; Lees et al., 2019, 2020) instead of boreal, where the growing conditions differ considerably from the temperate zone in terms of the length of the snow‐free season, and seasonal variations in solar irradiance and temperature. Additionally, much of the peatland surface vegetation studies in situ have been conducted using sensors that only record VIS‐ and NIR‐spectral regions (McPartland et al., 2019; Pang et al., 2022; Tucker et al., 2022). This means that the information regarding the species and the moisture content derived using data from the SWIR‐wavelength regions are currently unexplored for boreal peatland vegetation.

The aim of this study was to collect and analyze a spectral library of nine *Sphagnum* species common in European boreal peatlands and which form large growths, and that could, thus, be monitored with remote sensing sensors in the future. The moss samples were collected from natural, waterlogged peatlands after snowmelt. Using the newly collected spectral library, we (i) analyzed the intra‐ and interspecific variations in *Sphagnum* species' spectra in different moisture conditions and (ii) investigated how the moisture content of the mosses was related to their spectral properties. We also included key species whose spectral qualities have been studied very little, such as *Sphagnum centrale*, *Sphagnum girgensohnii*, *Sphagnum riparium*, and *Sphagnum rubellum*. This study has, to our knowledge, a larger sample size and a larger number of species than any previous study on *Sphagnum* spectra.

## 2 MATERIALS AND METHODS

### 2.1 Studied species

The *Sphagnum* moss species included in this study are common in southern boreal peatlands in northern Europe and represent a gradient of peatland nutrient levels. All studied species form homogenous growths that are large enough to be observed by high spatial resolution remote sensing sensors and, thus, are also large enough to be collected as samples for spectral measurements in a laboratory. All studied species form growths of at least 1 m^2^, whereas some species (e.g. *Sphagnum fuscum* and *Sphagnum cuspidatum*) can cover even over 10 m^2^ areas.

**FIGURE 1:**
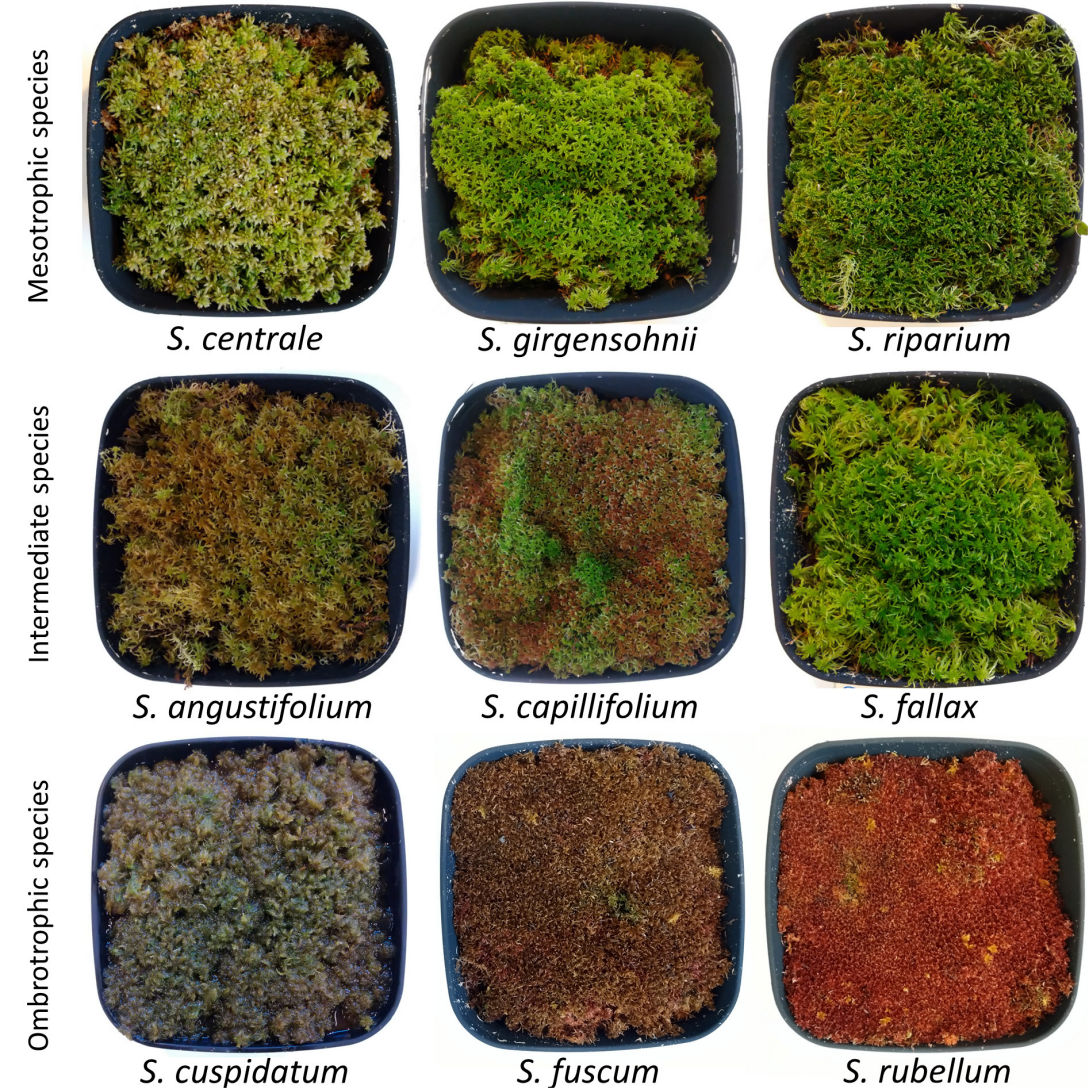
Photos of the studied species in the order of the nutrient status of the habitat. The species of the top row (*Sphagnum centrale*, *Sphagnum girgenshonii*, and *Sphagnum riparium*) are mesotrophic. The species in the middle row are intermediate, *Sphagnum angustifolium* representing oligotrophic and *Sphagnum fallax* the mesotrophic habitat, and *Sphagnum capillifolium* representing the dry ecotones of peatland and thus left outside of the habitat division. The species in the bottom row (*Sphagnum cuspidatum*, *Sphagnum fuscum*, and *Sphagnum rubellum*) are ombrotrophic.

A total of nine species were selected (Table 1, Figure 1). Three of them represent richer minerotrophic (i.e., mesotrophic) treed fen and spruce mire habitats (*S. centrale*, *S. girgensohnii*, *and S. riparium*), three of them represent intermediate fen habitats (*S. angustifolium*, *S. capillifolium*, *and S. fallax*), and three represent ombrotrophic bog habitats (*S. cuspidatum*, *S. fuscum*, *and S. rubellum*) (Figure 1). In the analyses, when the species were divided into two habitat groups, the mesotrophic *S. centrale*, *S. girgensohnii*, and *S. riparium* as well as the intermediate *S. fallax* comprised the mesotrophic group, and the ombrotrophic *S. cuspidatum*, *S. fuscum*, and *S. rubellum* as well as the oligotrophic *S. angustifolium* comprised the oligo‐ombrotrophic group. The last intermediate species, *S. capillifolium*, was left outside of the habitat division as it was collected from the ecotones, the edges of the peatland areas.

**TABLE 1:** The *Sphagnum* moss species selected for the study, as well as their nutrient status and habitat, in the descending order of nutrient level.

Species	Nutrient level	Description of habitats and habit of growth
*S. centrale*	Mesotrophic	Forms small cushions on spruce or deciduous‐dominated wooded fens
*S. girgensohnii*	Mesotrophic	Forms small mats or cushions in medium‐rich, tree covered fens or moist spruce forests
*S. riparium*	Mesotrophic	Forms large carpets in riparian habitats, e.g. along streams and ditches
*S. fallax*	Oligo‐mesotrophic	Forms large growths in poor to intermediate fens
*S. capillifolium*	Ombro‐oligotrophic	Forms hummocks and small lawns, can function as a paludifier for mineral soil
*S. angustifolium*	Ombro‐minerophic	Forms large growths or grows among other species
*S. cuspidatum*	Ombro‐oligotrophic	Forms large lawns and carpets on treeless bogs
*S. rubellum*	Ombrotrophic	Forms large, often homogenous lawns and carpets on treeless bogs
*S. fuscum*	Ombrotrophic	Forms large lawns and carpets on treeless bogs

### 2.2 Sample collection

The *Sphagnum* moss samples were collected from four peatlands in Uusimaa and Häme areas in southern Finland in May 2022 (Table 2). The sites were chosen based on the proximity to Aalto University, where the spectral measurements were conducted, to make the transportation time as short as possible. The sampling took place directly after the snow‐melting period, that is, all sampling sites were completely waterlogged during the sample collection. Each species was collected within 1 day (*S. angustifolium*, *S. capillifolium*, *S. centrale*, *S. fallax*, *S. girgensohnii*, and *S. riparium*) or two consecutive days (*S. cuspidatum*, *S. fuscum*, *S. rubellum*). The ~460 cm^2^ (21.5 × 21.7 cm) sized samples were collected by cutting the sample carefully using a shovel and scissors, and by trimming the dead *Sphagnum* from the bottom of the sample so that the depth of each sample varied between 5 and 7 cm. After the trimming, the sample was placed in an 8 cm deep measurement container, which had been painted black to prevent light scattering from the walls of the box during the spectral measurements (Figure 1).

**TABLE 2:** Sites where the samples were collected.

Peatland site	Coordinates	Collected species
Luutasuo	60.680°N, 24.321°E	*Sphagnum cuspidatum*, *Sphagnum fuscum*, *Sphagnum rubellum*
Matkunsuo	60.531°N, 24.710°E	*Sphagnum angustifolium*, *Sphagnum centrale*
Ritasaarensuo	60.640°N, 24.962°E	*Sphagnum girgensohnii*, *Sphagnum riparium*
Slättmossen	60.131°N, 24.365°E	*Sphagnum capillifolium*, *Sphagnum fallax*

A total of 10 living samples for each species were collected. To prevent multiple samples coming from one growth, the distance between each sampling location in a peatland site was at least 10 m. After being collected, sample containers were lidded and transported by car to Aalto University. The containers were unlidded promptly after the transportation and were kept open for the rest of the study. Before the spectral measurements, all litter on top of the moss, such as leaves or tree needles, was carefully removed with tweezers. The samples were measured for the first time within 4–6 h from the time they were collected.

Between the spectral measurements, the samples were stored in open containers in a semi‐dark room with no direct sunlight and standard temperature (average 20.5°C) and air humidity (average 29%). The samples were kept in the same container all the time to make sure that their structure would not be damaged during or between the spectral measurements.

### 2.3 Reflectance measurements and preprocessing of spectra

Reflectance measurements of the samples were conducted in a dark laboratory equipped for spectral measurements. The walls, doors, and ceiling of the laboratory were painted with black paint, and the measurement table was covered with optically black fabric. Each sample was measured four times: fresh (0 h), 1 day later (24 h), 2 days later (48 h), and 1 week later (1 week). The samples were photographed after the first and last spectral measurements.

Before each reflectance measurement, the sample was weighed with a standard scale. The dry mass of the samples was determined by weighing them 2 months after the spectral measurements so that they had been allowed to dry completely in the room where they were stored between the measurements. The dry mass was then used to determine the moisture content (Moist %) of each sample at the time of the measurement by first extracting the mass of the empty container and then applying Equation (1):

(1) $$\text{Moist}\%=\frac{F_{w}-D_{w}}{F_{w}}$$

where *F*_w_ is the mass of the sample at the time of the measurement, and *D*_w_ is the dry mass of the sample.

**FIGURE 2:**
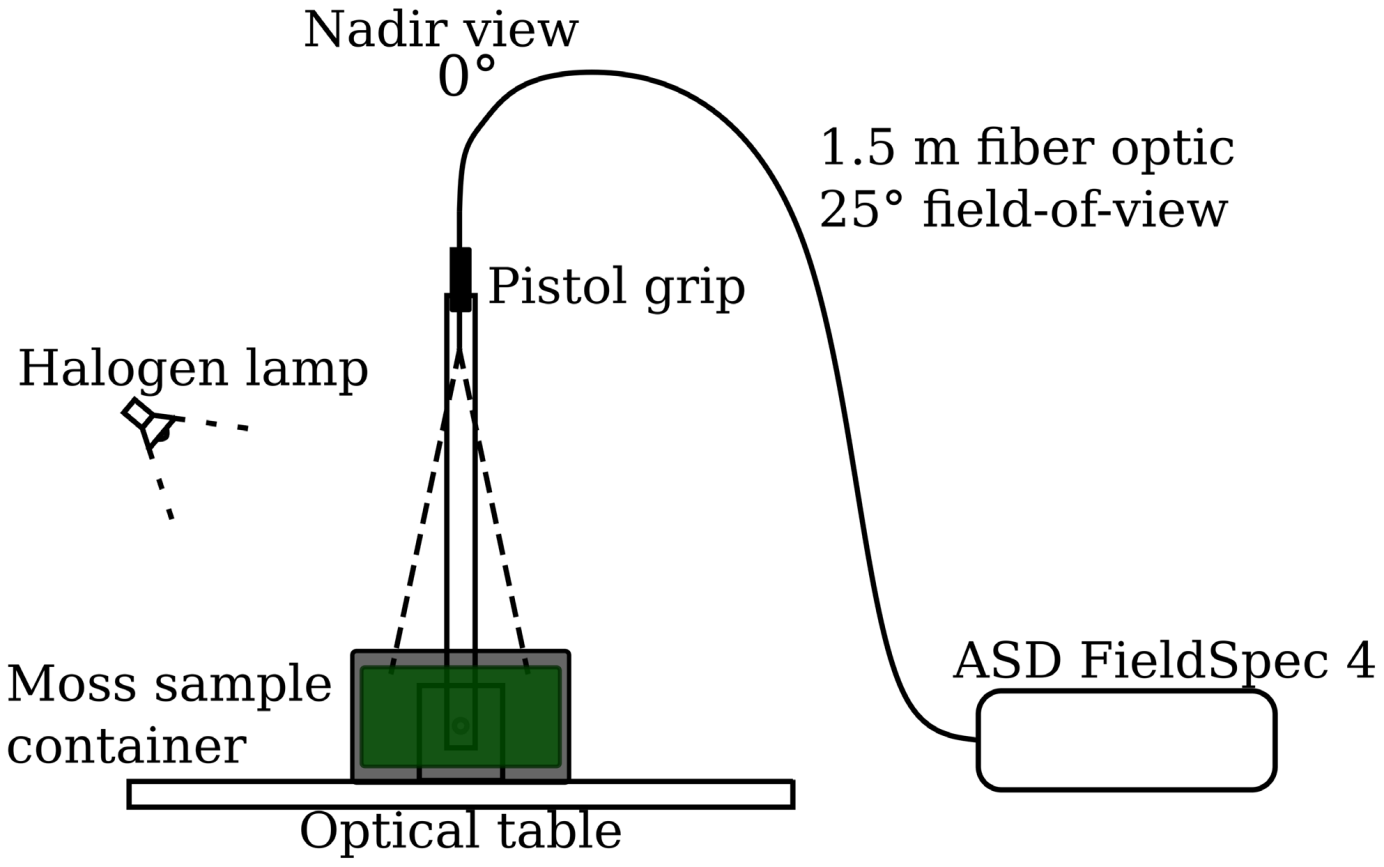
The measurement setup for the spectroradiometer.

Nadir‐view reflectance measurements were done with a FieldSpec 4 spectroradiometer (Figure 2) (Analytical Spectral Devices Inc (ASD), serial number 18641), which has a spectral range of 350–2500 nm, and its resolution at 700 nm is 3 nm and at 1400 and 2100 nm is 10 nm. The field of view was 25°. The measurement height was 30 cm, with the optical fiber of the spectroradiometer attached to a boom above the measurement table. The sample was illuminated with a 12 V 50 W Quartz Tungsten Halogen lamp with a 36° beam. The wide beam angle was restricted by fitting the lamp inside a custom black‐painted aluminum shade to diminish stray light from the surrounding surfaces. The zenith angle of the illumination was 40°. The container box of the sample was placed under the optical fiber, placing it carefully in the same position during each consecutive measurement time. Furthermore, the container was not rotated during the measurement repetitions.

The raw spectral data consisted of three repetitions of a measurement of an individual sample, which were then averaged as one. Similarly, at the beginning, end, and every 1 h from the start of a measurement session, white reference and dark current measurements were taken by repeating the measurement thrice and averaging the three measurements as one. The conical–conical reflectance factor (CCRF) (Schaepman‐Strub et al., 2006), hereafter called only reflectance, was calculated from them (Equation 2):

(2) $$\text{CCRF}=\frac{I_{s}-I_{\mathrm{dc}}}{I_{\mathrm{wr}}-I_{\mathrm{dc}}}*{\mathrm{RF}}_{\mathrm{wr}}$$

where *I*_s_ is the spectrometer reading of the sample, *I*_dc_ is the reading of the dark current measurement, *I*_wr_ is the reading of the white reference measurement, and RF_wr_ is the reflectance factor of the white reference. For the white reference, we used a 10‐inch factory‐calibrated Spectralon® reference panel with 99% nominal reflectance. After calculating the reflectance, the data were smoothed with a Savitzky–Golay filter (Savitzky & Golay, 1964) (25 nm window). Processing of spectral data was done with R software (R Core Team, 2022), with the R package hsdar (Lehnert et al., 2019). The processed spectra for all moss samples are openly available in Salko et al. (2023).

### 2.4 Data analysis

First, a mean spectral signature for each species for each measurement time was calculated by averaging the reflectance values over the 10 samples of each species. As the data were tested for normality and found not‐normally distributed, a Wilcoxon rank‐sum test was applied with a 5% significance level to compare the spectra of the first (0 h) and the last (1 week) measurement times for all samples of the same species. The purpose of the comparison was to examine in which wavelength areas the reflectances differ from each other statistically significantly. In addition, the position of the Red Edge Inflection Point (REP), a proxy of chlorophyll content (Miller et al., 1990), was calculated for each spectral signature, and the variance of the different species REP position was compared with a Wilcoxon rank‐sum test to examine whether REP has statistically significant differences between species.

Next, we analyzed how much of the spectral variation is explained by species or habitat. To achieve this, we used least squares estimation to fit a linear regression model (as described in Juola et al., 2022) where the reflectance was the response variable and (a) the *Sphagnum* species and (b) the habitat was the (categorical) explanatory variable. The result was quantified by computing and comparing the coefficients of determination (*R* ^2^).

To visualize the overall spectral variation between the species and to avoid multicollinearity and reduce the dimensionality in initial spectral data, principal component analysis (PCA) was conducted for the data collected in the first and last measurement times (i.e., 0 h and 1 week). From the PCA results and based on the first three principal components, Euclidean distance was used to calculate how the spectral data could be further grouped into smaller clusters. Here, we applied the hierarchical clustering analysis and dendrogram method. Both the PCA and the hierarchical clustering analysis were done with the R package FactoMineR (Lê et al., 2008).

Finally, we analyzed the relationship between the spectral properties and moisture content of the samples by using the Ratio Index (RI). From the spectral data, we calculated the RI for all possible wavelength combinations (Equation 3):

(3) $$\mathrm{RI}=\frac{R_{\lambda_{1}}}{R_{\lambda_{2}}}$$

where *R* is the biconical reflectance factor at wavelengths *λ*_1_ and *λ*_2_. The RI values were then fitted into a linear regression model, where the RI value was the response variable and the moisture % was the explanatory variable.

## 3 RESULTS

### 3.1 Reflectance properties of *Sphagnum* species

**FIGURE 3:**
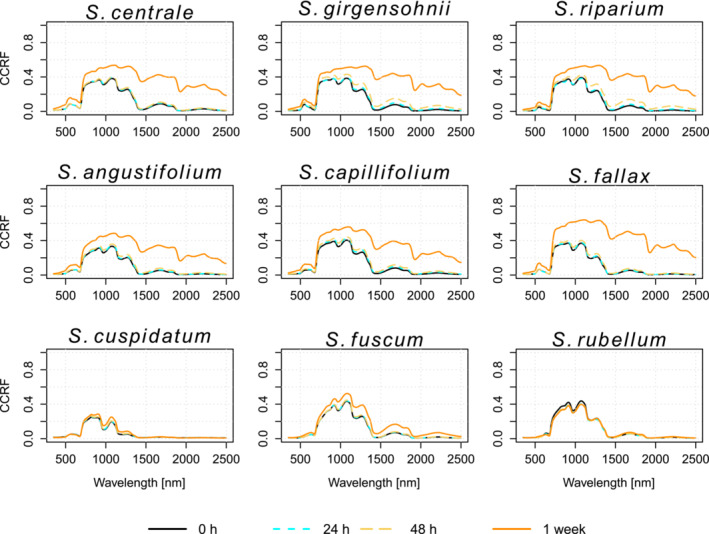
Mean reflectance spectra of each *Sphagnum* species at all measurement times. The species in the top row are mesotrophic, the species in the middle row are intermediate and the species in the bottom row are ombrotrophic.

The spectral signatures of the fresh *Sphagnum* mosses exhibited general features typical to green vegetation in the VIS and NIR regions but often had very low reflectance in the SWIR region compared with vascular vegetation (Figure 3). As the moss became drier, it also became whitish in appearance, which resulted in higher reflectance at especially NIR and SWIR wavelength regions (Figure 3). The only species that started to appear whitish before the 1‐week measurement was *S. riparium*, while *S. fallax* had the highest reflectance of all during the final measurement. Throughout the experiment, *S. cuspidatum* had the lowest reflectance of all species, being close to zero in VIS and SWIR regions.

**FIGURE 4:**
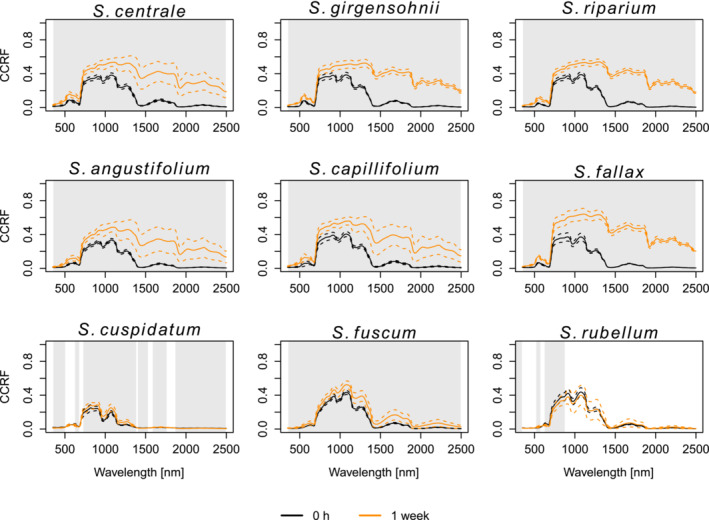
Mean spectral reflectance of the measured *Sphagnum* species (solid line) and the standard deviations of them (dashed line). Gray areas denote the wavelength regions with statistically significant differences between the measurement times. The species in the top row are mesotrophic, the species in the middle row are intermediate and the species in the bottom row are ombrotrophic.

For the first 2 days after being collected (i.e., corresponding to three measurement times), the mean reflectance spectra remained similar for all species (Figure 3). However, after drying for 1 week, the mesotrophic and intermediate species had much higher reflectance in the VIS, NIR, and SWIR regions than what they had during the first 2 days. Statistically significant differences in the reflectance spectra between the fresh (i.e., 0 h measurement) and 1‐week‐dried samples were observed for all mesotrophic and intermediate species throughout the entire spectrum (Figure 4). The reflectance of the ombrotrophic species, on the other hand, was either slightly higher (*S. cuspidatum* and *S. fuscum*) or marginally lower (*S. rubellum*) after 1 week of drying. Only for the ombrotrophic species, there were spectral wavelengths where the difference between the fresh and the 1 week dried samples' reflectance was not statistically significant. For *S. cuspidatum*, those wavelengths were in 508–619, 682–722, 1388–1399, 1534–1583, and 1766–1863 nm, for *S. fuscum* in 350–353 nm and *S. rubellum* in 350–351, 356–493, 597–611, 878–1896, and 2013–2039 nm (Figure 4). The within‐species variation in the spectral signatures was small in the beginning (0 h measurement) but became larger after 1 week (Figure 4) for each of the species except for *S. cuspidatum*, whose standard deviation was the lowest throughout the whole spectrum after a week of drying.

We observed some general spectral properties of the species that were connected to the nutrient level of the species' growing environment. Throughout the experiment, the mean reflectance of each species bore the most similarities with the rest of the species from its respective habitat. Ombrotrophic species (*S. cuspidatum*, *S. fuscum*, *S. rubellum*) had a distinguishably lower reflectance than the mesotrophic and intermediate species throughout the spectrum at all measurement times. The only exception to this was during 24 and 48 h measurements in the 900–1300 nm water absorption region, where *S. fuscum* and *S. rubellum* had similar reflectance to the mesotrophic and intermediate species.

**FIGURE 5:**
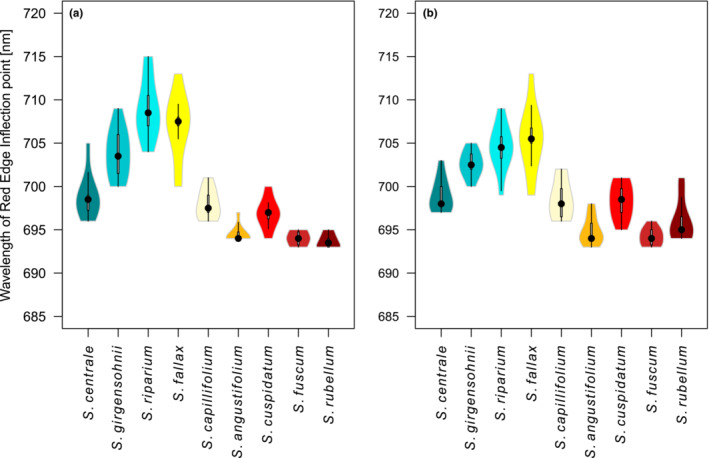
Red‐Edge Inflection Points of samples for (a) the 0 h and (b) 1‐week measurements. The blue‐tinged species are mesotrophic, the yellow‐orange‐tinged species are intermediate, and the red‐tinged species are ombrotrophic. The black dot signifies the median of the sample set.

Next, we looked at the REP of the species. Applying the Wilcoxon rank‐sum test with a 5% significance level revealed that the REPs of the species differed significantly from each other both during the 0 h and 1 week measurements (Figure 5). During the 0 h measurement, the REPs of each species could be grouped into (1) a mesotrophic group (*S. centrale*, *S. girgensohnii*, *S. riparium*, and *S. fallax*) with larger standard deviations and higher REPs and (2) an ombro‐oligotrophic group (*S. angustifolium*, *S. cuspidatum*, *S. fuscum*, and *S. rubellum*) with low standard deviations and mean REPs (Figure 5). After drying for a week, the mean REPs of the oligo‐ombrotrophic group were below 700 nm, whereas the mesotrophic group's REPs were mainly over 700 nm. For drier samples, there was also less within‐species variation in the REP (i.e., the standard deviation of the REP for each species was lower at 1 week than in the beginning of the campaign). *S. fallax* had the largest variation in REP in both times of measurements but more notably after 1 week of drying.

### 3.2 Relating species and habitats with reflectance data

**FIGURE 6:**
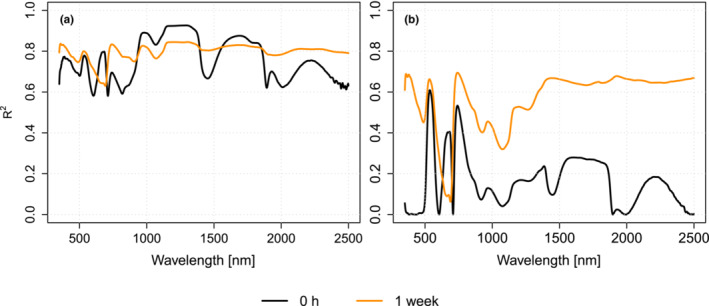
Coefficient of determination (*R* ^2^) of the linear model that explained the variation of reflectance, using (a) species and (b) habitat as the explanatory variable.

The results of fitted linear model indicated that species explained the variation in the reflectance more than the habitat (Figure 6). For example, in the freshly collected samples (i.e., the 0 h measurement), species explained over 90% of the variation in the 1100–1300 nm wavelength region. Habitat, on the other hand, explained, at maximum, 69% of the variation in reflectance spectra (at 740–743 nm) of the samples.

**FIGURE 7:**
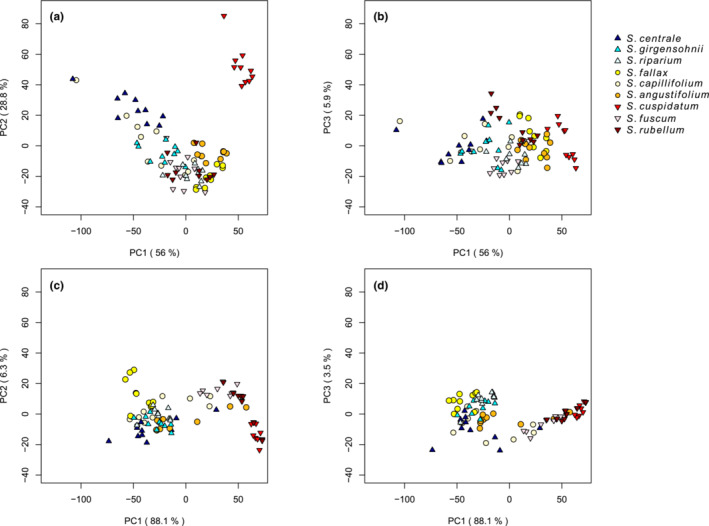
Projection of samples onto (a) the first and second and (b) the first and third principal components of the 0 h measurement, and projection of samples onto (c) the first and second and (d) the first and third principal components of the 1‐week measurement. The percentages of the total variance explained by each component are in brackets. Blue‐tinged triangles show mesotrophic species, yellow‐orange‐tinged circles show intermediate species and red‐tinged triangles show ombrotrophic species.

The first three principal components explained 90.8% of the variance in reflectance spectra of the fresh samples and 97.8% of the 1‐week dried samples (Figure 7). For fresh samples (0 h measurement), plotting principal component one (PC1) and principal component two (PC2) together showed that *S. cuspidatum* formed its own separate cluster, *S. centrale* and *S. capillifolium* formed two loose, overlapping clusters, and the rest of the species were clustered together (Figure 7a). Plotting together PC1 and the third principal component (PC3) showed that, for the fresh samples, the species formed mostly overlapping clusters, with *S. cuspidatum*, once again, as the only species with no overlap with the other species (Figure 7b). After 1 week of drying, the mesotrophic and intermediate species formed loose overlapping clusters in the PC1–PC2 space, and ombrotrophic species formed overlapping clusters with some overlap with the intermediate species and little overlap with the mesotrophs (Figure 7c). Plotting PC1 and PC3 for the same measurement time, on the other hand, interestingly revealed two more clearly separate areas, one where the mesotrophic and intermediate species mostly clustered, and one where the ombrotrophs clustered (Figure 7d).

**FIGURE 8:**
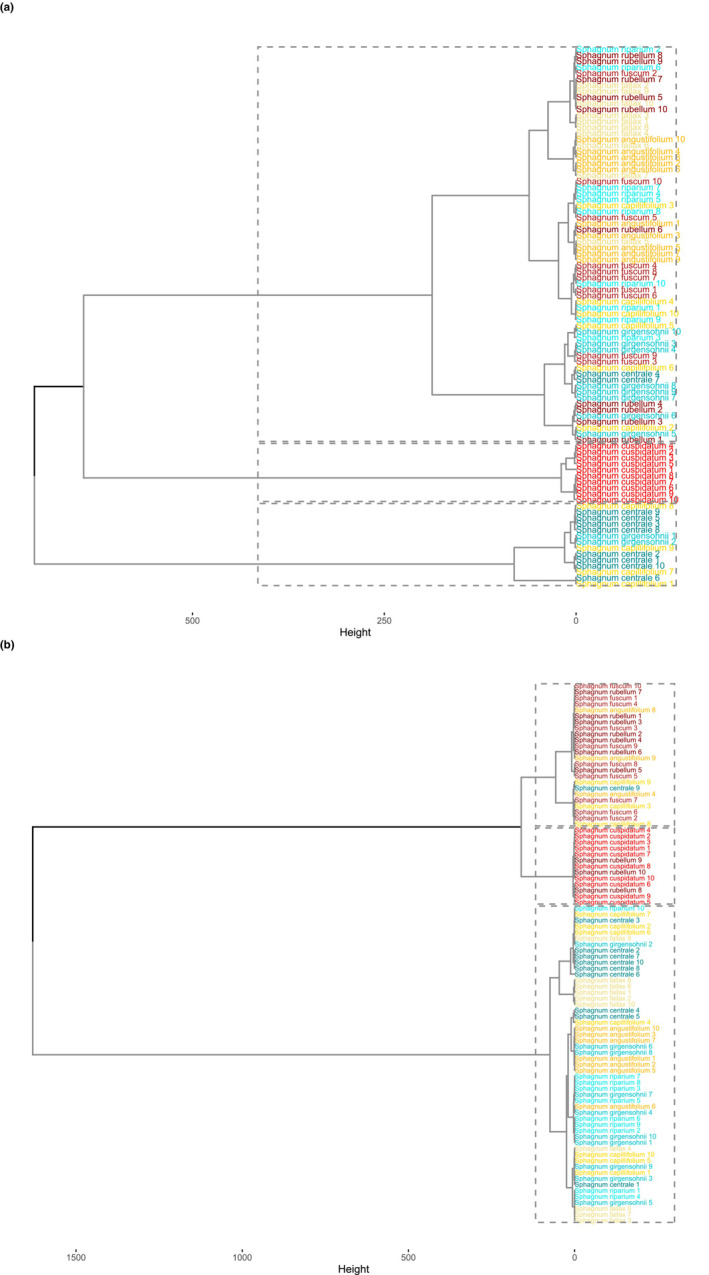
Dendrograms produced by hierarchical clustering analysis of (a) the 0 h measurement and (b) the 1‐week measurement. The blue‐tinged species are mesotrophic, the yellow‐orange‐tinged species are intermediate, and the red‐tinged species are ombrotrophic.

Finally, the first three principal components that explained most of the spectral variation were used as input variables for the hierarchical clustering analysis, which was used to examine which species are spectrally closest to each other. Because three principal components explained the majority of the variation in the spectral data in both fresh and 1‐week measurements, the number of clusters in the dendrogram was set to three. In the clustering analysis conducted with the fresh samples, three groups emerged: *S. cuspidatum* formed its own, pure group with all the samples in the cluster (Figure 8a). *S. capillifolium*, *S. centrale*, and *S. girgensohnii* formed another group with few exceptions of some samples of those species not clustering in it, and ultimately, the rest of the species clustering in the final group, often close to their respective species, with no apparent regard for habitat (Figure 8a). However, after 1 week of drying, the meso‐ and ombrotrophic groups emerged prominently, with nearly no mixing together, and *S. cuspidatum* clustered together with *S. rubellum* (Figure 8b). The intermediate species were scattered between the two clusters, and again, all species were clustered close to their respective species.

### 3.3 Estimating moisture content with reflectance data

The samples' mass and moisture content varied between the species, the mesotrophic species being the lightest and the visually sparsest and the ombrotrophic species being the heaviest as well as visually densest (Table 3). During the experiment, the mesotrophic *S. girgensohnii* and *S. riparium* lost the greatest amounts of their moisture out of all the species and the ombrotrophic species lost less of their moisture content than the other species (Table 3).

**TABLE 3:** The moisture content of the samples during the four measurements times. “Moist %” refers to the moisture content and “Mass, g” to the mean mass of each species.

	0 h		24 h		48 h		1 week		Dry	
	Moist %	Mass, g (standard deviation, g)	Moist %	Mass, g (standard deviation, g)	Moist %	Mass, g (standard deviation, g)	Moist %	Mass, g (standard deviation, g)	Moist %	Mass, g (standard deviation, g)
*S. centrale*	91.6	525 (125.7)	90.3	462.2 (131.1)	89.2	419.2 (132.8)	74.3	190.5 (96.6)	0	43.3 (8.2)
*S. girgensohnii*	87.1	371.2 (66.2)	85.2	325.3 (63.5)	81.5	261.7 (58.8)	49.3	98 (28.6)	0	47.6 (6.5)
*S. riparium*	88.9	422.5 (73.6)	87.2	366.9 (66.6)	83.8	291.7 (60.2)	64.4	136.9 (38.4)	0	46.2 (6)
*S. capillifolium*	89.9	616 (165.6)	88.4	541.1 (159.8)	87.4	508.6 (164.9)	73.1	258.1 (123.9)	0	60.8 (11.3)
*S. fallax*	91.6	588.2 (152.7)	90.5	523.8 (150.5)	88.7	449.7 (153.5)	73.9	208.2 (96.9)	0	48.3 (8.6)
*S. angustifolium*	90.3	763.4 (176.9)	89.5	712.6 (180.2)	88.2	638.3 (184)	80.9	406.5 (150.7)	0	72.2 (9.7)
*S. cuspidatum*	98.7	2646 (155.6)	98.6	2527.4 (173.6)	98.6	2507.8 (161.4)	98.4	2242.9 (136.6)	0	35.1 (9.4)
*S. fuscum*	92.6	1119.4 (163.5)	91.9	1026.4 (158.4)	91.6	991.8 (159.8)	87.4	677.9 (148.1)	0	81.7 (7.2)
*S. rubellum*	94.4	1611.7 (286.2)	94	1512.4 (273.6)	93.8	1476.8 (278.7)	92.2	1193.3 (289)	0	88.3 (6.3)

**FIGURE 9:**
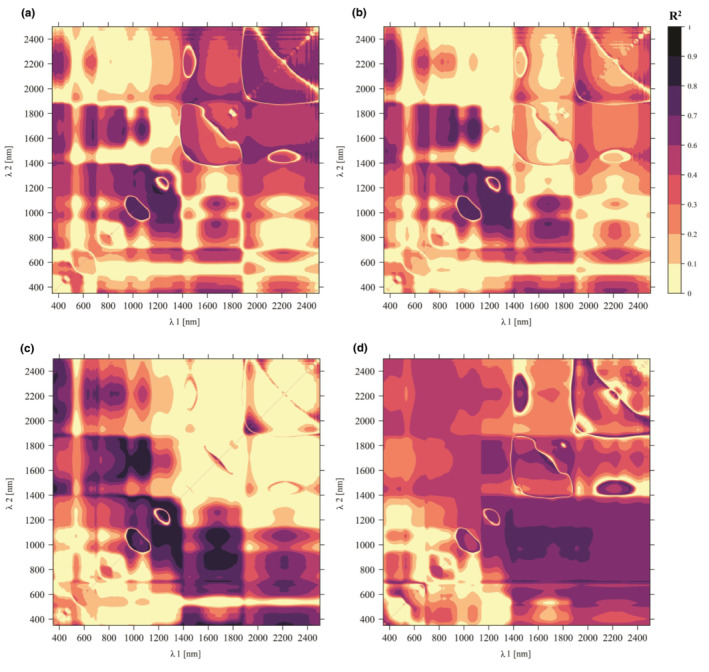
Heatmap of the coefficients of determination (*R* ^2^) for the moisture content of the sample and Ratio Index with all possible wavelength combinations (*λ*_1_, *λ*_2_ in the region 350–2500 nm) for (a) 0 h measurement, (b) 24 h measurement, (c) 48 h measurement, and (d) 1‐week measurement.

**FIGURE 10:**
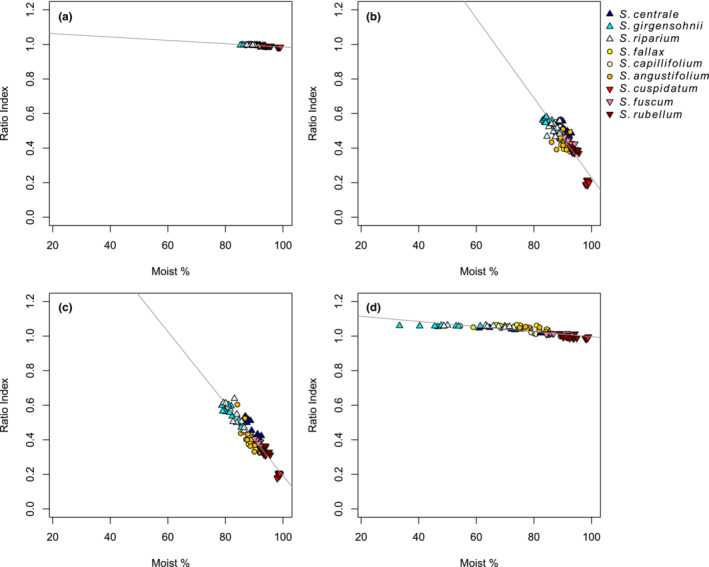
Linear regression model between the moisture content and the Ratio Index with the highest coefficient of determination in the moisture analysis for (a) 0 h measurement, (b) 24 h measurement, (c) 48 h measurement, and (d) 1‐week measurement. Blue‐tinged triangles show mesotrophic species, yellow‐orange‐tinged circles show intermediate species and red‐tinged triangles show ombrotrophic species.

Across all study species, the Ratio Index analysis demonstrated that the most informative bands for detecting moisture content of the samples were located in the 1100–1900 nm wavelength region, consistently for all four measurement times (Figure 9, Table 4). In the 1‐week measurements, the spectral regions, mainly in SWIR, that were strongly related to moisture content were considerably wider than for the fresh samples. The relationships between the RI and moisture content were strong (*R* ^2^ > 0.7) in the *λ*_1_ = 1200–1400 nm, *λ*_2_ = 1000–1200 nm regions throughout the first three measurements (Figure 10).

**TABLE 4:** The linear regression models explain the Ratio Index with the moisture content with the highest coefficient of determination.

	*λ*_1_ [nm]	*λ*_2_ [nm]	Linear model	*R* ^2^	RMSE
0 h	1197	1188	−0.001*x* + 1.081	0.790	0.002
24 h	1325	1095	−0.023*x* + 2.534	0.776	0.050
48 h	1342	1119	−0.021*x* + 2.276	0.889	0.039
1 week	1630	1729	‐0.001*x* + 1.144	0.720	0.014

The regions where the relationship was mainly weaker (*R* ^2^ < 0.2) were, for the 0 and 24 h measurements, in the VIS wavelengths and for the 48 h measurement, in the *λ*_1_ = 500–1000 nm, *λ*_2_ = 1900–2500 nm regions. For the 1‐week measurement, the regions with high *R* ^2^ were generally wider than in the first three measurements, *R* ^2^ ranging from 0.5 to 0.7.

## 4 DISCUSSION

### 4.1 Reflectance properties of *Sphagnum*

The nine *Sphagnum* species we analyzed were, to different extents, distinct from each other in their reflectance spectra. They were also visually distinct from the reflectance properties of vascular plants (e.g., Girard et al., 2020; Hovi et al., 2017; Meireles et al., 2020) and lichens (e.g., Kuusinen et al., 2020). Furthermore, the spectral responses the species had for desiccation were, especially for the mesotrophs, characteristic of their respective habitats.

Between‐species differences of the spectra were the smallest during the 0 h measurements, at mainly VIS wavelengths and generally increasing after 700 nm toward 1400 nm wavelengths. A similar observation was made for the 24 and 48 h measurements. During the 1‐week measurement, the between‐species variation was high throughout the whole spectral range, increasing in the SWIR region from 1000 nm onward. We speculate that many of the between‐species spectral differences could be due to the different biochemical compositions of the species, yet the lack of literature on *Sphagnum* mosses' biochemistry does not allow linking typical biochemical and spectral features, similar to what has been done for vascular species in peatlands (Girard et al., 2020). Overall, the reflectance of the species that have been explored in the previous studies, such as *S. capillifolium* and *S. fallax*, were similar in shape but a little lower than what Bubier et al. (1997) and Bryant and Baird (2003) have reported. The reflectance spectrum of *S. cuspidatum* reported by Bryant and Baird (2003) was both lower and of a different shape, suggesting that the samples we measured were considerably wetter.

As our study had a larger sample size per species than previous studies on *Sphagnum* spectra, we were also able to analyze intraspecific (within‐species) variation in the reflectance spectra of different species. Our results showed that, for the fresh samples, intraspecific spectral variation was small, but after 1 week of drying, the variation increased for all the mesotrophic and intermediate species, especially in the NIR and SWIR regions. For the ombrotrophic species, the effects of drying were not as distinctive, which suggests that they are less susceptible to desiccation during a short‐term, such as 1 week, drought.

While the sensitivity of *Sphagnum* mosses' spectral reflectance qualities to changes in moisture has been used in airborne mapping of peatlands' moisture conditions (Harris et al., 2006; Kalacska et al., 2018) and photosynthetic activity (Arroyo‐Mora et al., 2018), to‐date no study applying aerial hyperspectral monitoring of peatlands has attempted to separate the *Sphagnum* species from each other or to map species‐specific coverages in a peatland area. However, as our results indicate that *Sphagnum* species differ from each other spectrally, such applications could be possible in the future. Furthermore, as the different *Sphagnum* species also indicate different ecohydrological conditions of their growth environments and respond to moisture stress differently, applying species‐specific knowledge into hyperspectral mapping of peatlands would enable more accurate and comprehensive assessments of these ecosystems.

### 4.2 Relating species and habitats with reflectance data

Species explained over 90% of the variability in spectra of the fresh samples in 1135–1341 nm wavelengths. This underlines the importance of having access to species‐specific spectral libraries of *Sphagnum* mosses, also extending beyond traditional VIS–NIR data to longer NIR or SWIR wavelengths, when mapping species distributions in peatlands using high spatial resolution remote sensing data.

The habitats, on the other hand, explained clearly less of the spectral variation, exceeding 65% explicability at 352–402, 525–543, 1388–1575, 1856–2154, and 2321–2500 nm wavelengths during the 1‐week measurement. For the fresh measurements, the habitats usually explained less than 50% of the spectral variation. This is likely due to the high interspecific variation of the ombrotrophic species: they do not resemble each other at any point of the experiment as much as the mesotrophic species resemble each other. During the measurement of fresh samples, the species that had the most similar spectral properties with each other were from the mesotrophic habitats. Conspicuously, after 1 week of drying, the mesotrophic species became whitish in their appearance and resembled each other also spectrally. The ombrotrophs, and to some extent, the oligotrophic *S. angustifolium*, did not undergo such dramatic natural bleaching during the week of spectral measurements. An important mechanism behind this phenomenon was probably that the ombrotrophic species had a higher mass and their surface was much denser in appearance than the mesotrophs'. With less surface area than what the mesotrophs have, the ombrotrophs can restrict their evapotranspiration (Bengtsson et al., 2016; Hájek et al., 2009; Laing et al., 2014). This is also strengthened because of the structure of their pending branches which form the capillary network (Hayward & Clymo, 1983; Rydin, 1985). This is likely due to the less layered structure of their growth environments: the tree‐covered mesotrophic habitats provide more shade for the undergrowth mosses than the treeless ombrotrophic habitats (Laing et al., 2014). In our experiment, the species that are more used to the extreme conditions in their natural habitats could uphold their moisture for a week, and the species that came from more stable environments could not.

### 4.3 Estimating moisture content from reflectance data

The spectral combinations that were strongly associated with the moisture content were, even more notable than in the other analyses, within the SWIR region. Previous research has established that information regarding the peatland moisture conditions, and more specifically, water table depth, can be retrieved from peatland surface vegetation from the SWIR wavelengths and used in large‐scale monitoring of peatland water table depth dynamics (Burdun et al., 2020). The results of the *Sphagnum* moisture content analysis here support the results of the studies conducted with satellite data on peatland moisture conditions. This also highlights the importance of including the SWIR wavelengths when detecting moisture from peatland vegetation.

The relationships between the moisture content and reflectance data were the strongest after 2 days of drying (*R* ^2^ > 0.8). We also observed that when the drought proceeded, the SWIR region most strongly associated with moisture moved toward longer wavelengths (Table 4). During this measurement time, for some of the species, the samples were already partially dried while other species were not showing effects of desiccation. The spectral signatures' high sensitivity to the changes in moisture suggests that spectral data on *Sphagnum* mosses can be used as indicators of the peatland surface moisture content.

## 5 CONCLUSIONS

In this study, we examined the intra‐ and interspecific spectral properties of European boreal peatland mosses. We demonstrated that much of the spectral information regarding the *Sphagnum* species and their state of desiccation can be found in the longer NIR and SWIR region. We also reported to‐date the most extensive measurements on intraspecific spectral variation in *Sphagnum* mosses, showing that ombrotrophic species have very little intraspecific spectral variation as opposed to mesotrophic and intermediate species, which had much larger spectral variation within species. Further studies should assess the variability of the *Sphagnum* spectral properties during different states of the growing season, as well as with different genera of peatland moss species associated with eutrophic‐rich fens. Data from the new and forthcoming hyperspectral satellite missions, such as EnMAP, PRISMA, and CHIME, will also include SWIR bands, and thus, interpreting data collected by them will need to be based on a fundamental understanding of the spectral properties of peatland surface vegetation not only in VIS and NIR but also in SWIR. The *Sphagnum* moss spectra (350–2500 nm) measured in this study are publicly available (Salko et al., 2023). In the future, our spectral library can be applied for different purposes, such as in developing new remote sensing methods for mapping boreal *Sphagnum* mosses.

## AUTHOR CONTRIBUTIONS

**Sini‐Selina Salko:** Conceptualization (equal); data curation (lead); formal analysis (lead); investigation (lead); methodology (equal); visualization (lead); writing – original draft (lead). **Jussi Juola:** Investigation (equal); writing – review and editing (equal). **Iuliia Burdun:** Conceptualization (equal); methodology (equal); writing – review and editing (equal). **Harri Vasander:** Investigation (equal); writing – review and editing (equal). **Miina Rautiainen:** Conceptualization (equal); funding acquisition (lead); methodology (equal); project administration (lead); resources (lead); supervision (lead); writing – review and editing (equal).

## FUNDING INFORMATION

The study was mainly funded by the Academy of Finland (grant: PEATSPEC 3341963). This study has also received funding from the European Research Council (ERC) under the European Union's Horizon 2020 research and innovation program (grant agreement No. 771049). The text reflects only the authors' view, and the Agency is not responsible for any use that may be made of the information it contains.

## CONFLICT OF INTEREST STATEMENT

The authors declare there are no competing interests.

## Data Availability

The data are available in Mendeley Data (https://doi.org/10.17632/wm5fcxdmzd.1).
